# The promotion of critical reading through the digital environment: A study on the virtual epitexts used to promote children’s picturebooks

**DOI:** 10.3389/fpsyg.2023.1154513

**Published:** 2023-04-06

**Authors:** Rosa Tabernero-Sala, María Jesús Colón-Castillo

**Affiliations:** Department of Specific Didactics, University of Zaragoza, Zaragoza, Spain

**Keywords:** virtual epitexts, book trailer, critical reading, critical reader, children’s picturebooks, promotion by publishers, promotion of reading, non-fiction book

## Abstract

The most recent research studies in the field of reading describe a new cultural ecosystem in which analog and digital reading coexist and contribute to transform what is read, either through the way reading is performed or by promoting reading. In this context, the training of critical readers is particularly important, an aspect emphasized by UNESCO and the curriculum frameworks based on its premises. In order to provide data for reflection on this question, this paper presents an essentially qualitative and interpretive documentary study of a sample of 836 virtual epitexts that promote children’s picturebooks. The selected documents consist of the postings by 45 publishing houses between 2020 and 2022 on their YouTube and Vimeo channels. The results of the content analysis present the current tendencies in digital promotion of children’s books and the strategies most likely to encourage critical reading. The insistence on the author’s presence, the emphasis on the materiality of the book as an object, the strengthening of artistic discourse and the hybridization of reality and fiction, among other aspects, all propose a type of reading that favors the development of critical thinking. The results are complemented by a selection of virtual epitexts suggested to mediators and readers as resources of interest in promoting critical reading in socio-educational contexts.

## 1. Introduction

The coexistence of analog and digital paradigms in today’s information society has established a new reading ecosystem built on hybridization. This is evidenced in research into reading habits cited by scholars such as Cordón-García (2018, 2020) or Lluch (2018). The paradigm shift in reading has led to significant changes in the way children and young people approach information, knowledge and literature (Lluch, 2018). Social media have further influenced access to reading by throwing literate practices into an environment of constant and rapid modifications (Cruces, 2017; Lluch, 2017, 2018, 2021; Cordón-García, 2018). Contrary to expectations, the progress of digital publishing has strengthened the material aspect of reading to such an extent that the physicality of the book as an object has become one of the key elements in the new ecosystem, since, as Littau (2006) states, the format determines both the content and the layout.

In light of the challenges the digital society poses to reader training, the research conducted by Wolf (2018) is of particular interest. In her study, she defends the construction of a biliterate reading brain as the way we read influences the way we think. Wolf (2018) understands that the different digital experiences modern readers use—social media, games, interactive platforms—distance them from the in-depth reading connected to the development of critical thinking. The author, therefore, suggests a paradigm that combines analog and digital cultures to develop biliteracy from the beginning, with an emphasis on analog means in the early years to ensure access to the digital environment with guarantees at a later stage. The 2020 report by the Centro Regional para el Fomento del Libro en América Latina y el Caribe (Cerlalc) *Lectura en papel vs. lectura en pantalla* (Kovač and Van Der Weel, 2020) follows along the same lines. In one of its chapters, Støle (2020) develops the concept of in-depth reading by insisting on the conditions this entails, since reading on a screen, in her opinion, is inferior in terms of comprehension as digital media require less attention. In fact, as the Cerlalc Report (Kovač and Van Der Weel, 2020) indicates, difficulty in reading extensive texts rises as the number of personal digital devices available to children increases (Støle, 2020). This idea is further confirmed by the results of the report *Developing Literacy Skills in a Digital World* OECD (2021), presented by the OECD and based on the 2018 Pisa Report. Likewise, after reviewing 54 studies, Delgado et al. (2018) state that printed text is associated with better comprehension as paper reading requires greater concentration compared to screen reading (Gil-Pelluch et al., 2020). Furthermore, Schilhab et al. (2020) emphasize the sense of stability that printed paper bestows on a physical book, making it the most suitable medium for the development of in-depth reading. The European Commission report on the creation of a Work Plan for Culture (2015–2018), *Promoting Reading in the Digital Environment* (Unión Europea, 2016), and the report by the Federación del Gremio de Editores en España (Millán, 2017), among others, agree on this idea. Both reports provide robustness to this line of research, which emphasizes one of the challenges posed by the new ecosystem regarding the training of readers who are capable of selecting and organizing information to convert it into knowledge. In short, the digital society offers a new definition of the agents involved in the new reading ecosystem. Thus, the concept of the reader as prosumer (García-Roca and De-Amo, 2019; De Amo and García-Roca, 2021), the new role played by mediation (Lluch, 2017, 2021; Zafra, 2017; De Amo Sánchez-Fortún, 2021), reflection on the authorial paradigm (Unsworth, 2015; Tabernero-Sala, 2019; Tabernero-Sala et al., 2022) and the entity that virtual epitexts acquire in the promotion of reading (Lluch et al., 2015; Lluch, 2018) define the need to experiment with reading models that help to train critical readers who are able to cope with the tension between digital and analog cultures, as recommended in the studies by Baron (2015, 2021) and the exploratory research by Mizrachi et al. (2018) and Mangen et al. (2019), among others.

In this context, one of the challenges that the information society faces regarding reader training is none other than how to incorporate critical citizens into the new cultural ecosystem—citizens who possess the necessary strategies to express themselves using their own judgment in the midst of digital and analog paradigms (De Amo Sánchez-Fortún, 2021).

In the field of education, the reports by UNESCO insist on the need to develop critical thinking as a cornerstone in the construction of democratic, participatory societies capable of collectively meeting the challenges of the twenty-first century (Caro-Valverde, 2018). This is also expressed in the report *Reimagining our Futures Together: A New Social Contract for Education*—commissioned by UNESCO and prepared by the Comisión Internacional sobre los Futuros de la Educación, 2022. This report aims to promote lifelong education as a collective project, backed by the commitment to human rights, democratic participation and care for the planet. These objectives advocate for the questioning of absolute truths by citizens and, in doing so, they establish an education system that guarantees the access to accurate information as a basis for a commitment to truth.

In Spain, Organic Law 3/2020, of 29 December, which amends Organic Law 2/2006, of 3 May, on Education (España, Cortes Generales, 2020) expands on UNESCO indications and supports training that encourages critical thinking in primary and secondary education. Furthermore, Royal Decree 157/2022, of 1 March, which establishes the organization and minimum teaching requirements for primary education (España, Ministerio de Educación y Formación Profesional, 2022), introduces the concept of critical thinking linked to the training of competent, autonomous, critical readers who can understand and interpret multimodal texts that help them to meet the challenges of the modern information society. Media and information literacy is considered for this purpose, since its teaching focuses on information search strategies, acknowledgment of authorship, handling different documentary sources and the comparison, organization, critical evaluation and creative communication of information. In this context, the studies by Ennis (2011, 2018) and Kuhn (2018, 2019), among others, are of particular interest.

Ennis (2011, 2018) defines critical thinking as the reasonable, reflective thinking a person adopts regarding a subject on which they need to take some kind of decision. In this sense, citizens should be required to possess the abilities of a critical thinker, such as being well-informed, supporting beliefs on truths, justifying decisions and presenting reasons including points of view that differ from their own, while considering others’ feelings.

Kuhn (2018, 2019) understands argumentation as the axis around which critical thinking gravitates and as a fundamentally collective and contextualized social and cultural practice that should be reflected in the socio-educational context, as suggested by Guzmán-Cedillo and Flores-Macías (2020) in their review of 73 studies conducted between 2000 and 2016 in educational settings. In this regard, Bezanilla et al. (2019) have reviewed the main methodologies used to foster critical thinking and suggest that teachers in these settings should employ strategies such as posing questions, choosing activities with a real-life context, using different information sources, stimulating reflective dialogue, referring to visual displays and analyzing the arguments formulated by the community and research. Likewise, Witarsa, and Muhammad (2023) propose a learning model based on inquiry, discovery and problem-solving.

Children’s picturebooks are particularly appropriate for developing reading literacy in the early years, the stage when the analog paradigm should be prioritized, since, as the main research studies show (Wolf, 2018), in-depth reading, which is essential for developing critical thinking, is linked to physical books. From this perspective, an analysis of the evolution of the picturebook toward what is called ‘non-fiction’ (Grilli, 2020, 2021; Tabernero-Sala, 2022; Tabernero-Sala and Laliena, 2023) in reader training is required, as the non-fiction book occupies one of the most vital sectors in the publishing market (Jan, 2021) and has influenced the development of children’s books as a whole (Tabernero-Sala, 2022). In this respect, researchers are beginning to investigate the natural pairing of critical reading and the non-fiction picturebook (Trigo-Ibáñez et al., 2022). This type of children’s book is now the most interesting genre in children’s picturebooks, both nationally and internationally (Smith and Robertson, 2019; Dindelli, 2021; Jan, 2021), due to its artistic nature (Grilli, 2021); it has also become the reading model presented in fictional discourse aimed at children (Goga, 2020; Goga et al., 2021; Kümmerling-Meibauer and Meibauer, 2021).

In keeping with the line begun in the studies by Sander’s (2018) relating to the analysis of non-fiction picturebooks in connection to critical reading, focusing on the analysis of virtual epitexts as one of the strategies to promote reading is appropriate insomuch that we consider it important to analyze whether mediation fosters critical reading and, if applicable, which strategies the mediator should identify in order to recognize the promotional discourses that favor the development of the critical reader.

Social media have prompted the appearance of public, virtual epitexts that promote reading and have been generated by an inherent need for dissemination of publishing projects. Research studies such as those by Grøn (2014); Lluch et al. (2015), Basaraba (2016); Tabernero-Sala (2016b,2018), Tabernero-Sala and Calvo-Valios (2016), Ibarra-Rius and Ballester-Roca (2017); Rovira-Collado (2017), Dimova et al. (2018), Lebrun et al. (2018), Romero-Oliva et al. (2019, 2020, 2023), and Bilushchak et al. (2020) emphasize the entity acquired by virtual epitexts used in the promotion of reading, both in disseminating and in proposing reading models that they defend by identifying a new type of social reader who makes essential changes to the construction of discourse. The twenty-first century reader receives information on multimedia devices that involve multimodal discourse. Similarly to Gray (2010), Lluch et al. (2015) emphasize the need to reflect on the importance of these types of promotional epitexts that, far from being auxiliary, paratextual elements—in the sense of the definition of paratexts suggested by Genette (2004)—actually create the text, are part of its identity and determine the meaning that the reader assigns to it by proposing the reading strategy as a type of ‘textual consumption’ (Lluch et al., 2015). In earlier research works (Tabernero-Sala, 2016b), we analyzed in more detail the nature of the book trailer as one of the most significant virtual epitexts, both from the perspective of the promotion of reading and regarding its potential in training the readers of the twenty-first century, in such a way that it may even have its own artistic entity, in accordance with what Unsworth (2015) terms ‘multimodal literary narratives’. In some cases, it may, in fact, become a way of bringing interpretive rewriting closer to the reader (Tabernero-Sala, 2021). Therefore, analysis of virtual epitexts is vitally important to make a detailed study of the new methods of book promotion and how these methods—insofar as they create meaning and guide reading—determine the receiver the discourse requires. Thus, as a means of promoting reading and books—insofar as they create meaning—virtual epitexts offer the possibility of mediated reading that, in the case of children’s books, involves a reading model with links to the context in which it occurs, so much so that in previous studies (Tabernero-Sala et al., 2022) we have investigated the connection of the book trailer as an epitext for book promotion that defines a reading model in the case of non-fiction picturebooks. We understand that virtual epitexts may have changed in recent years, particularly since the COVID-19 health crisis, when, as several reports prove (Cencerrado Malmierca and Yuste Tuero, 2020; ERI-Lectura, 2020; Sánchez-Muñoz, 2022), reading habits changed and the use of virtual environments reached an unprecedented magnitude.

On this basis, we believed that an updated study on the virtual epitexts of children’s picturebooks would be of interest. Our initial proposal was to investigate and reflect on the new tendencies in the digital promotion of children’s books and to analyze to what extent, in the new cultural ecosystem, the promotion of reading in the area of children’s publishers shows a commitment to training critical citizens, in keeping with the UNESCO reports enshrined in different educational laws. Therefore, using the theoretical framework described and, in line with the studies by Gray (2010), we hereby present this study on the virtual epitexts used to promote children’s picturebooks, with a particular focus on the book trailer as the most widely used and firmly established tool for virtual promotion in the digital environment. The research concentrates on the following objectives:

Objective 1. To define the current tendencies of the virtual epitexts used in the promotion of children’s picturebooks.

Objective 2. To identify the construction strategies in virtual epitexts that are likely to encourage critical reading.

Objective 3. To select a corpus of examples of virtual epitexts as a resource for the promotion of reading and the training of critical readers in socio-educational contexts.

## 2. Materials and methods

To achieve the research objectives, we conducted a documentary study based on the content analysis of a sample of 836 pieces of digital audio-visual material. These audio-visual documents or videos were published by 45 publishing houses specializing in children’s literature from 1 January 2020—an important year due to the COVID-19 pandemic—to 31 December 2022. The main function of this digital content was to promote the reading of picturebooks aimed at children, so they are considered virtual epitexts of the books (Tabernero-Sala, 2016b).

**FIGURE 1:**
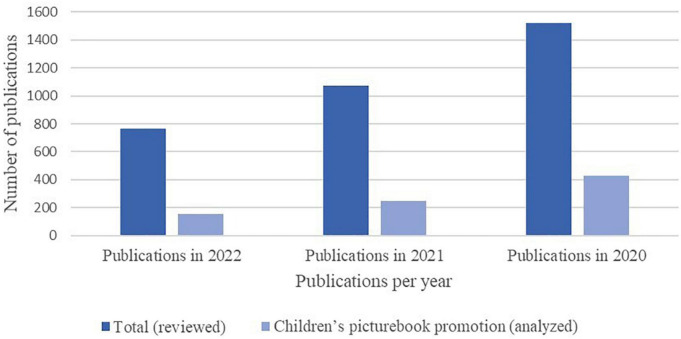
Audio-visual documents published on YouTube or Vimeo by publishers.

The process of selecting the 45 publishing houses and the 836 virtual epitexts consisted of three stages:

• Firstly, 68 publishers were selected as being of interest for the study. The main sources consulted in this stage were the directory of Spanish publishers specializing in children’s and young people’s books published by the Federación de Gremios de Editores de España, n.d., the publishing houses belonging to Asociación Álbum, n.d. and the Biblioteca Nacional de España, n.d. of specialist publishers. For this initial selection, two essential assessment criteria were considered: publishing in Spanish and being well-known publishers in the area of children’s literature.
• The second stage consisted of a review of the digital promotion methods used by the publishers in 2020, 2021 and 2022. The selection of the years responded to the first objective of the study—to define the current tendencies in digital promotion—and we were also interested in observing how these tendencies have evolved since the beginning of the COVID-19 pandemic. Different studies (Cencerrado Malmierca and Yuste Tuero, 2020; Sánchez-Muñoz, 2022) have explored how the pandemic has hindered access to books due to the closure of bookshops and libraries during 2020 or to social distancing, which affected reading in classrooms. We decided to analyze how publishers developed digital promotion of children’s picturebooks at that time, since, as shown in Figure 1, the number of publications on YouTube and Vimeo in 2020 increased. As for the promotion methods, we confirmed that the main, most stable method, which acted as content repository, was YouTube (Tabernero-Sala et al., 2022), and, to a lesser extent, Vimeo. Furthermore, publishers generally ran promotion on different social media, such as Instagram or Facebook, due to the immediate nature of these media and their great capacity for dissemination. The publications uploaded onto these media, however, posed problems related to their registration, such as frequent repetitions within the same document and instability. At the same time, we also observed that videos previously hosted on YouTube and Vimeo were usually disseminated through these media, so the study eventually focused on these two platforms. In this way, the final selection of publishers was adjusted according to the presence of the publisher on YouTube or Vimeo between 2020 and 2022. Table 1 shows the quantitative data of the selection process and the adjustment of the publishers selected. The sample was reduced from 68 to 45 publishing houses as some of the 68 publishers initially selected did not have a YouTube or Vimeo channel, others that did have one had not used it in the period from 2020 to 2022. These were, primarily, small, independent publishers, representing projects run by one or two people, usually involving a more limited capacity for media outreach. Additionally, certain large publishing houses had, in fact, used these channels but they had no audio-visual content aimed at promoting children’s picturebooks; in other words, some of the publishers selected had also published books for adult readers, so, in some cases, their promotional videos focused solely on this sector of the population, and consequently, they were excluded from the selection process if they did not have any promotional videos for children’s books. Table 2 presents the 45 publishing houses that were finally included in the research.

**TABLE 1:** Process of selecting publishers.

Publisher selection	Number of publishers
Initial selection of publishers specializing in children’s literature	68
Publishers without a YouTube or Vimeo channel	15
Publishers with a YouTube or Vimeo channel without any activity between 2020 and 2022	7
Publishers with a YouTube channel with activity between 2020 and 2022	44
Publishers with a Vimeo channel with activity between 2020 and 2022	2
Publishers with a YouTube or Vimeo channel with activity between 2020 and 2022, but without any publications of interest for the purpose of this study	1
Final selection of publishers (YouTube or Vimeo channel and publications relevant to the purpose of this study between 2020 and 2022)	45

**TABLE 2:** Publishers in the study sample.

1	A buen paso	16	Edelvives	31	Litera
2	A fin de cuentos	17	Ediciones Castillo	32	Lóguez
3	Akiara books	18	Ediciones El Naranjo	33	Maeva
4	Algar	19	Ekaré	34	Nórdica
5	Amanuta	20	Flamboyant	35	Nube Ocho
6	Anaya	21	Fondo de Cultura Económica	36	Nuevo Nueve
7	Andana	22	Galimatazo	37	Océano Travesía
8	Apila	23	Iamiqué	38	Penguin España
9	Babel Libros	24	Impedimenta	39	Pequeño Editor
10	Bookolia	25	Juventud	40	Pintar-Pintar
11	Carambuco	26	Kalandraka	41	Silonia
12	Coco Books	27	Kókinos	42	Takatuka
13	Combel	28	Lata de sal	43	Tecolote
14	Cuatro Azules	29	Libre Albedrío	44	Wonder Ponder
15	Diego Pun	30	Libros del Zorro Rojo	45	Zahorí Books

**FIGURE 2:**
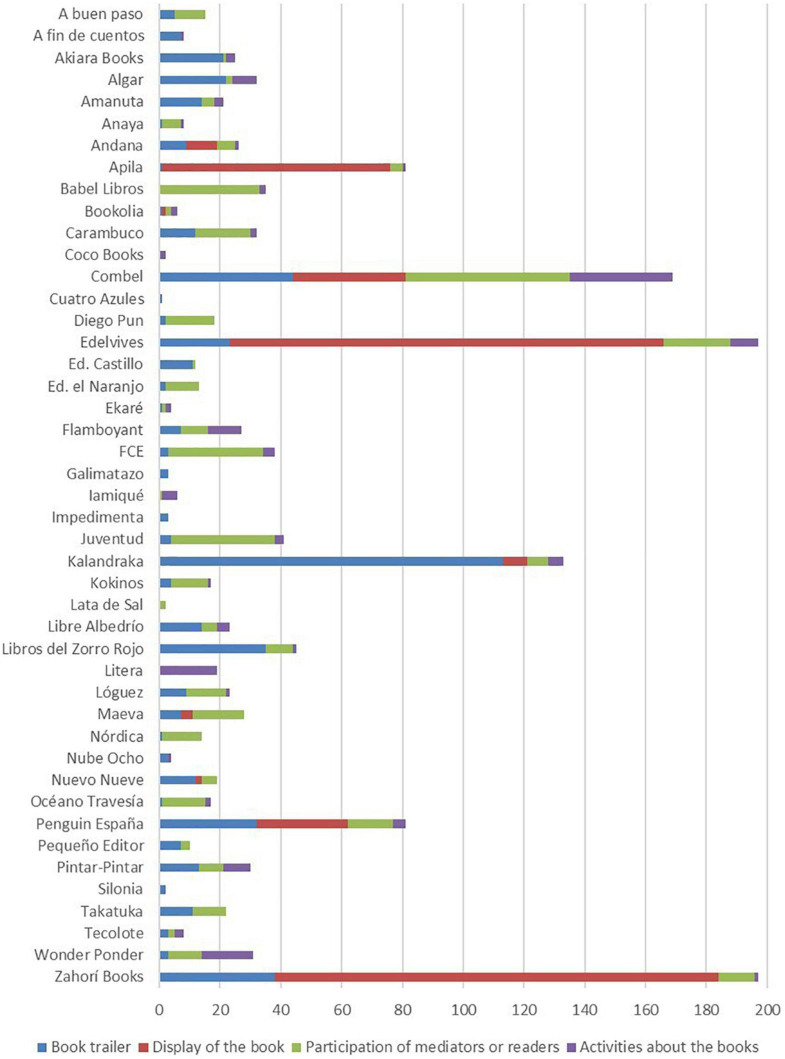
Type of videos for the promotion of children’s picturebooks selected, by publisher.

• The third stage of the document selection process consisted of a review of all the audio-visual publications on the official YouTube and Vimeo channels belonging to the 45 publishing houses—3,358 documents in total. This review identified the virtual epitexts used in the promotion of children’s picturebooks. Through this process, other types of content by some publishing houses were dismissed. The videos that were discarded from the study were those that promoted textbooks intended for the context of formal education or books aimed at young adult or adult audiences; we also discarded videos that presented pedagogical and educational conferences or videos that promoted other merchandising products, such as toys or videogames. In turn, videos aimed solely at the promotion of children’s picturebooks, whether they were works of fiction or non-fiction, were included in the selection, excluding textbooks; we selected book trailers with a cinematographic style, videos displaying the book as an object that only showed the book or part of the book, videos in which writers, editors, book-sellers, librarians, readers or other mediators presented, commented on or read the book, or videos that showed activities or the publishers celebrating an event about this type of book (Figure 2); in other words, this study included audio-visual content aimed at promoting children’s books, although we found that the promotional styles and the strategies used were varied and they merged together, thus revealing some significant tendencies. The analysis of this aspect was of interest to this study and the results are presented below. Lastly, considering these criteria, 836 audio-visual documents were selected for study. Table 3 lists the audio-visual documents published each year by the group of 45 publishers and the number of virtual epitexts that were selected for the study.

**TABLE 3:** Audio-visual documents reviewed and documents selected.

Audio-visual documents reviewed		Audio-visual documents selected for analysis (virtual epitexts)	
Total publications in 2022	765	Publications selected from 2022	157
Total publications in 2021	1,078	Publications selected from 2021	252
Total publications in 2020	1,518	Publications selected from 2020	427
Total publications reviewed	3,361	Total publications analyzed	836

Once the selection process had been completed, each publisher’s digital channel and the videos published were systematically registered arranged by date. This stage involved the registration of the quantitative data of interest for the research objectives and for the content analysis (Krippendorff, 2019). These data included the date of creation of the channel, the number of subscribers, the number of total views on the channel, the date each video was uploaded to the internet, the duration of each video, the number of views up to the date of the analysis and the corresponding links. Table 4 shows the registration sheet used for each of the 45 publishers.

**TABLE 4:** Register sheet for audio-visual documents.

Name of publisher		Register date of audio-visual data		
Name of YouTube or Vimeo channel	Channel creation date	Number of channel subscribers	Total number of channel views	Channel link
Number of total publications by publisher in 2022		Number of publications selected from 2022 whose objective is the promotion of children’s picturebooks		
Number of total publications by publisher in 2021		Number of publications selected from 2021 whose objective is the promotion of children’s picturebooks		
Number of total publications by publisher in 2020		Number of publications selected from 2021 whose objective is the promotion of children’s picturebooks		
Total number of publications by publisher		Number of publications selected for analysis		
**Register of each of the virtual epitexts selected (one row per video)**				
Video title	Date of video upload	Video duration	Video views	Video link

Following the registration of the audio-visual documents, a mainly qualitative content analysis was conducted focusing on the constructive and rhetorical strategies in the audio-visual discourse typical of digital book promotion (Basaraba, 2016). The content analysis was based on a process of categorization, coding, analysis, refining of categories and qualitative interpretation, using a narrative approach based on the postulates of the school of critical theory, as indicated by Ruiz-Olabuénaga (2009). The results of the analysis according to the research objectives are presented below.

## 3. Results

### 3.1. Current tendencies in digital promotion

As regards the first objective, the analysis of the audio-visual documents detected five emergent macrocategories. These macrocategories identify the most frequent components currently emphasized by the virtual epitexts analyzed and help to classify the videos according to their constructive model and to determine the main tendencies in digital promotion of children’s picturebooks. The macrocategories detected are listed and described in Table 5.

**TABLE 5:** Emergent macrocategories.

Macrocategories	Description
Emphasis on the materiality of the book	Videos focusing on displaying the book as an object, on page-turning and paratexts: illustrations, book covers, foldouts, pop-ups.
Emphasis on the video editing and the esthetic component	Animated and cinematic-style book trailers
Emphasis on the interpretation of the work and/or its construction process	Participation by authors, editors, booksellers and other adult mediators to present a book, provide comments or reflect on the creative process
Emphasis on the reading of the book	Reading aloud, songs, storytellers, oral narration.performed by adult mediators
Emphasis on the reception of the book and its receiver	Participation by child readers, activities suggested in the book, extension of the work.

**FIGURE 3:**
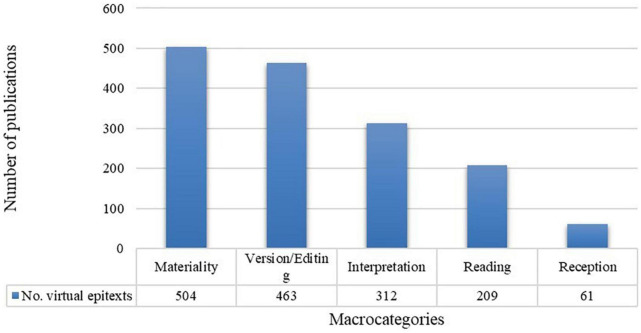
Macrocategories detected in the virtual epitexts.

Figure 3 presents, in absolute values, the incidence of these macrocategories in the virtual epitexts analyzed. The macrocategories are not mutually exclusive, as several may be present in the same video.

#### 3.1.1. Emphasis on the materiality of the book

Virtual epitexts that affect the materiality of the book is as a dominant tendency on some publishing channels. This type of video presents the book as an object, with an emphasis on its paratexts, mainly illustrations or other elements such as book covers, foldouts and pop-up components. Therefore, the picturebook aimed at children tends to be displayed as a promotional strategy, with an emphasis on its physical features, in contrast to other genres, such as the novel, predominantly focused on protecting the work. Promotion is based, therefore, on encouraging child readers to want to interact with the book and enjoy touching it, handling it and discovering what is hidden in its pages. The study identifies publishers that base promotion on a display of the book, such as Akiara Books, Apila, Edelvives and Zahorí Books. Other publishers have opted for this form of promotion at specific times, as was the case with Andana in 2021 or Penguin España in 2022.

Among the virtual epitexts that focus on the book as an object, differences can also be observed regarding their construction strategies. In this respect, videos usually concentrate on the turning of the pages: as the fast-motion turning of all the pages in the book employed by Apila or Nuevo Nueve; the leisurely page-turning used by Zahorí Books, Andana or some of the videos by Maeva or Tecolote; the page-turning that pauses to explore and handle the book, with zoom effects to emphasize the paratexts or certain content, such as in the videos by Edelvives; or some less frequent artistic approaches, such as some examples by A buen paso or Kókinos. Other publishers attempt to develop their own style, such as the recent publications by Penguin España, very short videos that present the book and some of its pages in a clearly edited format or the virtual epitexts from Akiara Books, which are contextualized in a natural environment and carefully edited as far as esthetics and the senses are concerned (Table 6).

**TABLE 6:** Examples of virtual epitexts based on the book’s materiality.

Book title	Publisher	Date of upload to channel	Duration	Views (to date indicated)
*¡Gracias, Madre Tierra!*	Akiara Books	09/12/2022	00:01:36	191 (11/01/2023)
Link	https://vimeo.com/779672069			
*Bajo el mar*	Penguin España	24/10/2022	00:00:29	62 (09/01/2023)
Link	https://youtu.be/prDtVgbiuJU			
*Reinos minúsculos*	Edelvives	15/11/2022	00:01:00	571 (06/01/2023)
Link	https://youtu.be/EoePirPE1A4			
*Bienvenida*	A buen paso	22/02/2021	00:01:03	777 (29/12/2022)
Link	https://youtu.be/RpwKjlPie68			
*¡ Ves lo que yo veo?*	Tecolote	30/09/2020	00:00:47	67 (03/01/2023)
Link	https://youtu.be/P4K9ujte2eM			
*Pompas de jabón*	Kókinos	23/12/2020	00:01:16	391 (11/01/2023)
Link	https://vimeo.com/494118535			

#### 3.1.2. Emphasis on video editing and the esthetic component

Animated book trailers, characterized by their cinematographic style (Tabernero-Sala, 2016b) and their emphasis on the esthetic component are among the virtual epitexts analyzed. The most outstanding examples stand out for replacing the still image, typical of a book, with a moving image, typical of the cinema, including a soundtrack and creating a story with its own artistic entity. These elements are usually linked to a storyline synopsis and address the reader directly through questions about the continuity of the story. These resources aim to create an intriguing context, arouse curiosity and thereby encourage reading of the book. These videos require careful, professional design and editing, which involves financial investment. Therefore, few publishing houses maintain the quality in this type of digital promotion, as it is increasingly being replaced by ‘homemade’ videos and live recordings without any editing. Publishers still opting for book trailers include A Fin de Cuentos, Kalandraka, Libre Albedrío and Libros del Zorro Rojo, whose videos are defined by their artistic quality. Other publishers such as Amanuta also used to publish professional animated book trailers, but have almost ceased to do so or use them exceptionally for certain outstanding works, as this type of video is not usually the main focus of their digital channel. This is the case of Akiara Books, Combel, Edelvives, or Zahorí Books. As for their duration, they last one minute on average, although there was a general tendency in 2022 to reduce the length of these videos—except for a few significant exceptions—possibly due to their dissemination on social media such as Instagram—where speed and brevity are the priority—and to their cost. Table 7 presents some animated and cinematic-style book trailers.

**TABLE 7:** Examples of animated and cinematic-style book trailers.

Book title	Publisher	Date of upload to channel	Duration	Views (to date indicated)
*La Reina de las Nieves*	Edelvives	20/12/2022	00:00:34	212 (06/01/2023)
Link	https://youtu.be/JztN1YIMLeg			
*Bibliotecarias a caballo*	A fin de cuentos	09/12/2022	00:00:36	13 (30/12/2022)
Link	https://youtu.be/gcb8BkdXsrg			
*Con esta línea*	Combel	04/11/2022	00:01:03	298 (30/12/2022)
Link	https://youtu.be/ib969TsDK8I			
*Circo*	Kalandraka	04/04/2022	00:02:22	197 (11/01/2023)
Link	https://youtu.be/S2vwwiHNb6g			
*Bajo las piedras*	Akiara Books	01/07/2020	00:01:58	721 (11/01/2023)
Link	https://vimeo.com/434288011			
*Travesía*	Libros del Zorro Rojo	09/06/2020	00:01:28	1,897 (29/12/2022)
Link	https://youtu.be/CZGkTGfPqb0			

#### 3.1.3. Emphasis on the interpretation or construction process of the book

The presence of authors and editors in virtual publications is a tendency shared Most of the publishers’ channels analyzed. The reason for their appearance may be to read the book, as we will see but, most usually, they appear to present books, promote reading, provide an interpretive commentary or reflect on the creative process. There are several different formats of virtual epitexts that emphasize these aspects according to, above all, the degree of complexity of the editing. On one hand, there are extensive formats, such as interviews, conferences and events. These videos are frequently recorded live with the participation of different guests or they even offer open access to the audience. Solo recordings of the authors commenting on their work are also common. The channels of the publishing houses A buen paso, Babel Libros, Combel, Diego Pun, Ediciones El Naranjo, Fondo de Cultura Económica, Nórdica or Wonder Ponder, among others, contain this type of publication, mainly intended for adult mediators, although content aimed at children—such as some videos by Combel—can also be found. The publisher Takatuka launched the initiative ‘Las librerías recomiendan,’ a series of videos in which different booksellers recommend and comment on a book by this publisher. The frequency of these publications is remarkable, once again, in 2020. They commonly contain references to the lockdown and the benefits of reading in this context. On the other hand, more elaborate formats requiring editing are also published, showing the authors’ workspaces or following the development of their creative process. This type of video is less common but publishers such as Andana, Edelvives, Pequeño Editor, Penguin España or Takatuka have some noteworthy titles (Table 8).

**TABLE 8:** Examples of virtual epitexts focusing on understanding and the creative process.

Book title	Publisher	Date of upload to channel	Duration	Views (to date indicated)
*Martín y la nuez inolvidable*	Fondo de Cultura Económica	16/11/2022	00:04:05	143 (08/01/2023)
Link	https://youtu.be/Lp1jEybJpXc			
*El Elefante*	Nórdica	29/04/2022	00:01:28	88 (03/01/2023)
Link	https://youtu.be/KEtTpOEd_IE			
*Tan solo un instante*	Edelvives	15/11/2021	00:01:17	5,838 (06/01/2023)
Link	https://youtu.be/nRWtaTr3Ipk			
*¡ Noche, toca los platillos!*	Pequeño Editor	02/08/2021	00:03:04	7,634 (03/01/2023)
Link	https://youtu.be/MuY46jHtE44			
*El destino de Fausto*	Andana	17/09/2020	00:02:13	1,338 (30/12/2020)
Link	https://youtu.be/TDSPN4JK6dc			
*La bañera*	Takatuka	09/07/2020	00:01:02	92 (03/01/2023)
Link	https://youtu.be/cXULdqSXCRw			

#### 3.1.4. Emphasis on the reading of the book

A marked tendency, mainly in 2020, is that of digital publications aimed at the reading of the book. In them, authors, editors, youtubers and other adult mediators read, sing or tell a story. A significant example is the ‘cuentacuentos’ (storyteller) initiative by the publisher Juventud, with 32 publications between April and July 2020. In these videos, the pages are displayed while a voiceover reads the text. Other publishing houses take the same line. For example, Carambuco provides publications both in an oral format and in sign language while displaying animated scenes from the book. In 2020 as well, Combel launched ‘cuentacombel,’ a project that offered, among others, readings of chapters of *Las aventuras de Pinocho* with the participation of different writers. Publishing houses such as Lóguez, Océano Travesía, Anaya, Fondo de Cultura Económica or Pintar-Pintar issued publications with the same purpose. On many occasions, the authors of the works or other mediators record the reading at home, as occurred during the lockdown. These initiatives did not continue after 2020, but they helped to boost a type of promotional video—characterized by its spontaneity—that has been used since then by many of the publishers analyzed. Furthermore, there are also edited virtual epitexts, of a more artistic nature, in which animation or the contemplation of the book are complemented by a voice telling part of the story, as is the case in some of the videos by Kalandraka or Ediciones Castillo. Table 9 provides examples of formats that emphasize the reading of the book.

**TABLE 9:** Examples of virtual epitexts focusing on the reading of the book.

Book title	Publisher	Date of upload to channel	Duration	Views (to date indicated)
*La pirata Guiomar*	Maeva	24/05/2022	00:08:46	42 (02/01/2023)
Link	https://youtu.be/fLOgNGC3jxA			
*Dos ositos*	Kalandraka	03/12/2021	00:01:35	243 (11/01/2023/)
Link	https://youtu.be/tOMsWpkXElI			
*Sol solito*	Libre Albedrío	19/03/2021	00:00:37	1,186 (09/01/2023)
Link	https://youtu.be/7sZw5Q3f2-c			
*Palmir*	Ediciones Castillo	05/02/2021	00:01:07	89 (02/01/2023)
Link	https://youtu.be/DPjgKAMeSe8			
*La oficina de objetos perdidos*	Juventud	01/05/2020	00:06:04	1,905 (30/12/2022)
Link	https://youtu.be/4CcIyS9cp24			
*¡ Qué lío cósmico!*	Carambuco	07/04/2020	00:10:09	1,593 (04/01/2023)
Link	https://youtu.be/zQCR7vJSTII			

#### 3.1.5. Emphasis on the reception of the book and its receiver

Another emerging tendency, a minority in quantitative terms but still of interest to this study, is the presence of child mediators who read, give opinions, recommend or interact with the books. In these videos, the publisher’s promotion relies on the receivers and peer dialogue, by trying to arouse empathy in the child audience. Similarly, some videos point out how to use a book, contain activities linked to the work or show its ludic and creative possibilities apart from reading it, even referring to other virtual epitexts, such as interactive games. These virtual epitexts emphasize the reception process of the book and offer replicable models for child readers; in other words, they provide ideas and proposals that extend the reading of the work and boost its potentiality. Again, there are two types among the publications showing this tendency. On one hand, there are more spontaneous videos, which consist of everyday recordings intended for immediate dissemination, for instance, some publications by Fondo de Cultura Económica or the publisher Pintar-Pintar, or the initiative by Wonder Ponder designed in 2020, which encourages children to participate by explaining how they felt during lockdown. On the other hand, although less frequently, there are more elaborate videos in which promotion is the underlying purpose. Their construction involves design, editing, animation and attention to the esthetics of the virtual epitext, as in some significant examples by the publishers Kalandraka or Zahorí Books (Table 10).

**TABLE 10:** Examples of virtual epitexts that emphasize the role of the child reader and extend the reception process.

Book title	Publisher	Date of upload to channel	Duration	Views (to date indicated)
*¿Quién soy? Crías de animales*	Kalandraka	29/07/2022	00:02:20	69 (11/01/2022)
Link	https://youtu.be/ykvn6oebpLY			
*Agua y Tierra*	Amanuta	16/05/2022	00:03:45	33 (30/12/2022)
Link	https://youtu.be/ZjbkKPIfox0			
*Ppprrrrriiit*	Zahorí Books	15/02/2022	00:01:05	125 (04/01/2023)
Link	https://youtu.be/3XGCXHWi2CA			
*Mi gran granja*	Lóguez	02/07/2021	00:02:40	56 (02/01/2023)
Link	https://youtu.be/gifZb79vr_M			
*Secreto de familia*	Fondo de Cultura Económica	09/04/2020	00:05:05	153 (08/01/2023)
Link	https://youtu.be/KxFy80fStLI			
*Etenko y los patines maravillosos*	Pintar-Pintar	07/04/2020	00:02:29	64 (04/01/2023)
Link	https://youtu.be/rdUnRsLDHWY			

### 3.2. Strategies for promoting critical reading

After exploring the current tendencies that define the virtual epitexts selected, the analysis then focused on identifying the discourse markers and strategies likely to encourage critical reading, while also considering the theoretical framework established previously. To this effect, a selective analysis of a qualitative and interpretive nature was conducted with the purpose of fulfilling study objectives 2 and 3; in other words, in this case, the interest of the study lay not so much in the frequency with which a specific discourse marker appeared in the virtual epitexts, but rather in identifying, in the wide range of publications previously described, the discourse markers present in the virtual epitexts most likely to promote critical reading. To this effect, we separated and analyzed the discourse markers that identified strategies that promoted a type of reading focused on cracks in the discourse, on questioning the sources and developing empathy as a method to raise awareness resulting from access to knowledge (Ennis, 2011, 2018; Kuhn, 2018, 2019; Bezanilla et al., 2019). The analysis was complemented by a selection of virtual epitexts with comments, which are suggested, ultimately, as resources that may be of interest to mediators and readers in promoting reading and training critical readers in socio-educational contexts. The virtual epitexts in the final selection promote books that should also be recommended for classroom reading in order to favor the development of biliterate critical reading by merging analog and digital formats (Wolf, 2018). Table 11 shows the discourse markers and strategies identified by this second analysis that are subsequently commented and exemplified.

**TABLE 11:** Discourse markers and strategies identified.

Discourse markers	Strategies for encouraging critical reading
Presence of the author of the book: voice of authority, opinion, reflection, subjectivity, questions for the reader.	The author is made visible as a metadiscursive strategy: this encourages reflection and questioning of the voice of authority
	Transmission of the author’s empathic projection on the discourse: this encourages empathy, understanding and awareness
Hybridization of fiction and non-fiction: scientific information, questions, fictional characters, plural testimonies, symbiosis between the natural world and the fictional world.	Intensification of experiential reading: links are created between the reader’s own experience and reading
	Strengthening of the relationship between the author, the reader, the book and the surroundings: curiosity and searching is encouraged, the relationship between the book and the surroundings is extended, through feedback that transforms the relationship of the individual with their context.
Emphasis on the materiality of the book: format, texts, illustrations, book covers, pop-ups.	Encouragement of exploration and the physical handling of the book, of enjoyment and detailed, in-depth reading
Presence of a narrator: voice, reading the book aloud, encouragement of reading, interpretation, guidance.	Proposal of models for reading aloud and oral narration: reading promoted as a sensorial experience
Discursive multimodality: texts, images, music, voice.	Proposal of a poetic and artistic discourse that addresses, moves, engages and interacts with the reader

#### 3.2.1. The visible author as a metadiscursive strategy

As confirmed by the tendency analysis, one of the recurring markers in virtual epitexts used for the promotion of children’s books is the presence of the author. Documents containing examples of this discourse marker can be found in promotions such as those by the publisher A buen paso for *Semillas*. *Un Pequeño gran viaje* by Alonso and Paschetta (2018) and *Zum zum. El viaje de la semilla* by Ferrada and Paschetta (2021). In this case, it involves a conversation between the editor and the illustrator of the two works regarding the creative processes.^1^ This provides an explanation about the universe in which the illustrator works according to the publisher’s proposal. Similarly, Jeffers (2019) appears in the book trailer for *El destino de Fausto*, a classic-style book trailer with sophisticated animation.^2^ Along the same lines, Pacheco (2020) explains his interpretation process regarding the work by Sister Juana Inés de la Cruz in *Caperucita Roja*, *Primero sueño*,^3^ Goes (2016) develops his creative activity^4^ or Dautremer (2021) demonstrates her creative process in her workspace, without saying a word, in *Tan solo un instante*.^5^ In these cases, the visibility of the people behind the books increases, which leads to the personalization of a discourse that does not come from the anonymity typical of children’s books, but rather from a discourse with its own ideology that corresponds to the author’s ‘world view.’ Therefore, whether fictional or non-fictional, the discourse is presented as a subjective proposal that responds to the bias of the person who has created it and that may, in this way, be questioned by the reader, who is required to form an opinion. More specifically, certain epitexts emerge as paradigmatic, such as those related to the recent promotion of two particularly relevant picturebooks: *Dos ositos* by Ylla (2021) and *Ernesto el elefante* by Browne (2021). The virtual promotion document of *Dos ositos*^6^ follows the style of a traditional book trailer in the first part---using a narrative voiceover with moving images of the illustrations in the foreground, which are photographs of the protagonists---and, the second, shorter part presents the author, her particular features, her life plan and her image contextualized in the time the work was created. Thus, the reader and mediator are presented with the directions the dialogue may subsequently take in the work, awareness of sustainable development and love of animals and the use of documentary images, which ensures the hybridization of reality and fiction. To a lesser extent, the promotional book trailer of *Ernesto el elefante*,^7^ incorporates the figure of the author to contextualize the proposal and suggest contemplation of the work within the framework of an entire authorial universe with an ideological bias.

On these lines, we recover a study by Sander’s (2018) on the connection of the non-fiction book with critical reading, since we understand that his research may apply, to some extent, to the analysis of children’s books, whether fictional or not, in a critical sense. In his study *A literature of questions. Non-fiction for the Critical Child*, Sander’s (2018) defines the genre as a literature of questions rather than answers requiring a critical reader to construct its meaning.

Sanders analyses the non-fiction book in terms of the presentation of information, of the bias authors place on the book simply by selecting some pieces of information to the detriment of others; he also investigates the esthetic strategies that invite readers to become involved in the texts, to interact with them, to engage with the information in a reflective, dialogic way. The intention is not to present information for it to be merely absorbed, but rather to encourage readers to investigate for themselves by arousing their curiosity and wonder in relation to what is presented.

These works propose critical readers who call into question what is presented to them, have an opinion on what they are shown, notice cracks in the discourse and question the credibility of the information. Based on ideas Barthe’s (1994) on the ‘death of the author’ and Freir’s (2002); Freire (2010) premises regarding critical pedagogy, along with Bakhtin’s (1981) dialogic concept of the novel, Sander’s (2018) identifies the critical reader in works with cracks in the authority of the text and questions that guide the process of intellectual inquiry. Therefore, centralization of meaning in a single, authorized source of true knowledge is questioned. Using the markers that Sanders identifies as the ‘visible author,’ the discourse presents the story using the voice, attitude and viewpoint of the author, who becomes part of the story through their ideas and opinions. Although, rhetorically, the interpretation may seem to be conditioned, Sanders bases his assertions on the research by Paxton and Zarnowski (Sander’s, 2018, p. 58), who showed, at the time, that readers’ interactive responses were more likely to occur when the author was present.

#### 3.2.2. Empathic projection of the author on the discourse

Along the same lines, with an emphasis on metadiscursive visibility, book presentation from an emphatic perspective acquires particular relevance. In this way, readers identify themselves through exposure to the authors’ own experiences, whether they are writers or illustrators. This is the case, for example, in the promotional epitext for *El patito feo* (Andersen and Abramović, 2021), in which the illustrator, Marina Abramóvic, describes her experiential process of reading Andersen’s (2021) work^8^ or the promotional epitext for *Muñeco de barro*, in which the illustrator, Carme Solé-Vendrell (Reyes and Solé-Vendrell, 2020), talks about her personal interpretation of the characters and their surroundings.^9^ Also relating to awareness-raising, the editor of Libros del Zorro Rojo discusses *Hablo como el río* by Scott and Smith (2021), suggesting the lines of dialogue the story opens.^10^

#### 3.2.3. Experiential reading: Between reality and fiction

In recent years, promotional virtual epitexts that defend removing the boundaries between fiction and non-fiction have taken the same direction. The intention is, ultimately, to relate reading to the reader’s personal experience and environment. Therefore, they show documents that encourage an emotional and physical connection between the books and the contents proposed. Thus, for example, in cultural and ideological terms, book trailers contain very clearly identified spaces and music. The book trailer for *¡ Gracias, madre Tierra!*, illustrated by Starkoff (2022), based on the Haudenosaunee Thanksgiving Address is a clear example.^11^ It is the translation, in foldout format, of a remarkable prayer offering a greeting and gratitude to all living beings, which has been recited every morning for many centuries by the whole community in a territory located between the United States and Canada. This defines the book as an inventory of the natural world and the wonders that nature offers. The natural backdrop against which the work unfolds establishes a relationship between the book and its physical surroundings by reflecting on fictional and artistic discourses and their natural extension along the lines proposed by the Sustainable Development Goals contained in the 2030 Agenda and the report *Reimagining our Futures Together: A New Social Contract for Education*, prepared by the Comisión Internacional sobre los Futuros de la Educación, 2022. The book trailer for *Tiempo de haikus* by Santaeulàlia and Lozano (2022)^12^ contains the same theme and promotional concept.

#### 3.2.4. The author, the reader, the book and the environment

In the register of experiential reading, there are promotional proposals that address the reader through questions raised directly by the works and generate interpretations of the setting by concealing the boundaries between the book and reality through the use of real scenarios where the act of reading occurs. An example of this proposal is the book trailer for the collection *¿Quién soy?* by Seceda and García (2021)^13^ where narrators’ and characters’ voices combine in open spaces in which fiction intertwines with readers’ realities and the contents are revealed as being part of receivers’ own lives. In this sense, the promotional epitext of *Brujas, guerreras y diosas* by Hodges and Lee-Merrion (2020) is paradigmatic, as it is a document structured around testimonies from female readers who have chosen stories about fairies, vampires, sorceresses and goddesses with whom they identify and present them as contemporary role-models. Therefore, the reader’s reality and the fictional discourse are connected, thus removing the boundaries between reality and fiction. The book trailer for the picturebook *¿Debo argumentar el sinsentido de la esclavitud?* by Frederick Douglass, with commentary by Squilloni and Fosch (2021) and translated by Jordi Pigem and Catarina Sacramento is of particular interest.^14^ It concerns the speech given by the ex-slave Frederick Douglass on Independence Day in the United States. The appeal to the reader, inherent in its ironic content, is made more powerful through the voices of the young black people who read aloud the first sentences of this rhetorical work of art while looking straight at the camera.

#### 3.2.5. From the materiality of the discourse to in-depth reading

Another of the discourse markers present in promotional virtual epitexts in recent years is the relevance that the physicality of the act of reading acquires through the conception of the book as an object. While in previous studies (Tabernero-Sala, 2016b,^2019^) we insisted on the tendency to emphasize the materiality of the book as one of the categories used to define the book trailer, in the last three years this category has become so usual that it has been identified as a dominant tendency, which may be explained by the digital environment with which people engage. Thus, book trailers such as those relating to the promotion of *Bienvenida* by Comín (2021a),^15^ *Mi arbolito de Navidad* by Comín (2021b),^16^ *Los volcanes* by Geis (2022)^17^ or *¡ No chupes este libro!* by Ben-Barak and Frost (2020)^18^ combine the dimension of the work as an object with specific references to its physical handling by the reader, at a leisurely pace that is a delight to the senses. This material dimension is part of the identification of in-depth reading to the development of the critical reader, involving the defense, as recent research recommends (Wolf, 2018; Zafra, 2019; Gil-Pelluch et al., 2020; Støle, 2020) of the time to think and memory retention that paper affords in contrast to the immediacy of the characteristic ‘skim reading’ of screens. The study conducted by Zafra (2017, p. 19), among others, refers to reading online every day as an experience based more ‘on impressions than on concentration,’ more on screenshots than on deliberate reflection. Zafra (2019) consequently defends the notion of ‘time to think’ as the only way to achieve autonomous, critical thinking.

#### 3.2.6. The sensorial experience. The narrator’s voice

This register also includes the importance attributed to the narrator’s voice by the different promotional epitexts, whether it is the actual author, writer/illustrator, or oral narrators who read aloud in spaces physically suited to the content of the book. There is an increased presence of documents in which the narrator’s voice becomes one of the key elements of the promotional proposal. In this manner, book trailers such as the one for the picturebook *¡ Artista!*, by Hernández-Sevillano and Cerro-Rico (2019),^19^ which presents a dramatized recitation of the text over the images, or the reading aloud of *Nunca dejes de brillar* by Alonso and Muñoz (2020)^20^ may serve as examples of pronounced discourse markers in the promotion of reading for children since the year 2020, closely linked to the necessity of defending reading as a sensorial experience.

#### 3.2.7. The multimodal discourse

Cinematographic book trailers that develop a multimodal discourse in which music, image, animation and voice produce artistic documents with an entity of their own follow the same direction (Unsworth, 2015). Thereby, book trailers such as those for *Bambi* by Benjamin Lacombe (Salten and Lacombe, 2020),^21^ *El bolero de Ravel* by Abad and Delicado (2020),^22^ *Shh*… *Tenemos un plan* by Haughton (2019)^23^ or *Abecedario* by Kaufman and Franco (2017), Letra Ñ^24^ use ellipsis, metaphors and appeals to the reader in their composition to move readers and interact with them.

## 4. Discussion

In terms of specialized publishers’ promotion of children’s books, the analysis presented reveals that, in recent years, virtual epitexts have evolved in proportion to the new challenges posed by the digital society regarding reader training (Cordón-García, 2018, 2020; Lluch, 2018). The data examined from the sample selected reveal an increase in the number of virtual documents created by publishers relating to the dissemination of children’s books in recent years, with the peak incidence rate in 2020, possibly due to the changes produced by the COVID-19 health crisis. This fact is consistent with the results of the reports on post-pandemic reading habits among young people and adults (Cencerrado Malmierca and Yuste Tuero, 2020; ERI-Lectura, 2020; Sánchez-Muñoz, 2022). Similarly, the presence of the book trailer as a document to promote reading has decreased in comparison to less elaborate, more immediate virtual materials, as required by social media, where the ephemeral prevails over anything requiring a longer production time with the consequent economic costs involved. In response to the speed that characterizes the digital society, only a few publishers have opted to attend to the esthetic quality of virtual epitexts and offer what Unsworth (2015) terms ‘multimodal literary narratives’ in their digital extensions.

As far as the tendencies observed are concerned, the five macrocategories defined in the initial descriptive analysis identify significant differences compared to the virtual dissemination scenario of previous years (Lluch et al., 2015; Tabernero-Sala, 2016b). Materiality emerges as the tendency with the greatest presence in virtual promotional documents, in keeping with developments in the field of children’s book publishing (Kümmerling-Meibauer, 2015; Tabernero-Sala, 2016a,^2019^), although this is unevenly reflected in the documents analyzed, as some of them are simply recordings of a reader handling a book. Since 2020, the voices of authors and readers fill the virtual epitexts used in book promotion, emphasizing the documentary nature of the act of reading and reinforcing the presence of creators, mediators and readers in line with the overuse of the testimonial literary practices typical of the digital culture (Zafra, 2019).

To answer the question concerning the connection between promotional digital epitexts and the proposal of critical reading, we understand that what we have termed the ‘visible author’ (Sander’s, 2018) can be defined as one of the most significant discourse markers in the virtual epitexts selected in the qualitative content analysis. In this sense, the presence of the author in all aspects establishes cracks in the concept of ‘truth,’ by offering the reader a personal and ideological relationship to what is explained or related, and shows the mediator, and consequently the reader, the possibility of establishing methods of argumentation and questioning to use as a basis for the development of critical thinking, through the integration of different viewpoints, as advocated by Ennis (2011, 2018) and Kuhn (2018, 2019). It is not surprising that this concept of the ‘visible author’ includes the figure of the editor, thereby establishing the concept of a joint authorship that constantly addresses the reader. In the same vein, the empathic projection of the ‘visible author’ paves the way to raising receivers’ awareness, involving, once again, the connection between emotion and knowledge, one of the most significant aspects in contemporary theories on non-fiction picturebooks (Grilli, 2020; Tabernero-Sala, 2022). Likewise, promotional virtual epitexts should emphasize and encourage the presence of discourse markers linked to the hybridization of fictional and non-fictional discourses through the use of different types of language, leading to, as the case may be, what we have termed experiential reading. In this way, the promotion reinforces the relationship between the reader, the book and reality by suggesting the connection of the book with reading environments in which shared natural settings are the priority, in line with the proposals contained in the Sustainable Development Goals. The artistic and experiential component extends the relationship between the book and its environment, through feedback that transforms the individual’s relationship to their context, as studied in the ecocritical paradigm of research on children’s books (Goga et al., 2018).

The emphasis on the materiality of the book and the presence of voiceovers, oral narrators and musical discourses aimed to arouse emotion and wonder, as well as curiosity, recover the sensorial aspect of reading, since the act of reading engages the body (Littau, 2006). With materiality as a basis, in-depth reading is identifiable in the concept of multimodal reading, which requires the reader to pause (Wolf, 2018; Zafra, 2019; Gil-Pelluch et al., 2020; Støle, 2020) for a time, which results in deliberation and enjoyment typical of artistic discourse.

In summary, virtual epitexts for the promotion of children’s picturebooks may provide mediators and readers with means, such as those revealed in the selection above, with which to develop the basis of critical thinking and meet, in this way, the challenge posed by the digital society in training readers within the framework of a biliteracy that has already been established (Wolf, 2018). Following on from previous studies (Tabernero-Sala et al., 2022), the selected virtual epitexts present discourse markers typical of the promotion of the non-fiction picturebook, thereby confirming, as reported by recent studies (Von-Merveldt, 2018; Grilli, 2020; Salisbury, 2020; Dindelli, 2021; Jan, 2021), the vitality of this genre that has changed the panorama of children’s books by defining a new reading model based on the hybridization of fictional and non-fictional discourses according to the contexts of the reader and the mediator.

## 5. Conclusion

The analysis of the results defines this research based on the following findings:

• Firstly, new tendencies resulting from the challenges posed by the digital society in recent years and new literate practices are described from the perspective of digital promotion (Cordón-García and Muñoz-Rico, 2023).
• Secondly, the results distinguish discourse markers that favor critical reading, which was previously non-existent in promotional virtual epitexts. The visibility of the author, the removal of the boundaries between reality and fiction, the experiential connection between the author, the reader and their environment, and the enhancement of the material aspects of the discourse all emphasize in-depth reading through the use of digital promotion (Wolf, 2018), along the lines established in the premises on critical reading advocated in international documents, such as *Reimagining our Futures Together: A New Social Contract for Education* (Comisión Internacional sobre los Futuros de la Educación, 2022).
• It is therefore understood that promotional virtual epitexts create meaning (Gray, 2010) and propose, from the digital paradigm, a way of reading that fosters the essential analogical aspect of in-depth reading, which is key to critical thinking. Consequently, a corpus such as the one selected for this research could be defined as a selection of recommended good practices in the context of training future mediators (Trigo-Ibáñez and Santos-Díaz, 2023; Álvarez-Ramos et al., 2023).

This opens future lines of research to delve into the analysis of the virtual epitexts used in the promotion of reading and into the reading paradigm that they present. Similarly, from this perspective, there is a need for research into how social media influence the contents and methods used in the promotion of reading in the digital society. Finally, the study of model virtual epitexts should continue to transfer the results to reading mediators.

## 6. Limitations

This research was essentially conducted in the context of Spanish publishing, although some Latin American publications were also selected, since the main criterion was for the works to be published in Spanish. Therefore, we consider that the sample of 836 audio-visual documents is representative for the research objectives but the results are not generalizable to other contexts. Furthermore, the audio-visual documents in the sample have been taken from two digital media, YouTube and Vimeo, given that other social media replicated content and were more unstable; this means that some publishers who had abandoned these platforms may have produced significant publications via other digital media on the dates indicated that have not been registered. Regarding the method used, the approach is quantitative as regards data registration and detection of the initial categories, by frequency and saturation, and qualitative-interpretive as regards content analysis and reporting of results. As a consequence, the limitations involved in research with these characteristics, concerning representativeness or generalization, should be assumed in the interests of the reflection and in-depth analysis required by the objectives of this study as a contribution to educational research.

## Data availability statement

The original contributions presented in this study are included in the article/supplementary material, further inquiries can be directed to the corresponding author.

## Author contributions

RT-S: conceptualization, analysis, writing of the manuscript, review, and supervision. MC-C: methodology, analysis, editing, review, and supervision. Both authors contributed to the article and approved the submitted version.

## Footnotes

1
https://youtu.be/UBU0B9GKy_g

2
https://www.youtube.com/watch?v=TDSPN4JK6dc

3 https://youtu.be/mB9YTBW-N2s; https://youtu.be/WyRzjtFfa-Y; https://youtu.be/lx0H8n0rqYI

4
https://www.youtube.com/watch?v=2Ig4JPGWLWw

5
https://www.youtube.com/watch?v=nRWtaTr3Ipk

6
https://www.youtube.com/watch?v=tOMsWpkXElI

7
https://www.youtube.com/watch?v=i860KJY0h9Y

8
https://www.youtube.com/watch?v=fK4dVVAL45g

9
https://www.youtube.com/watch?v=sDp_ZioYU-4

10
https://www.youtube.com/watch?v=OPQpuzlv_Ho

11
https://vimeo.com/779672069

12
https://vimeo.com/738779606

13
https://www.youtube.com/watch?v=ykvn6oebpLY

14
https://vimeo.com/657981925

15
https://www.youtube.com/watch?v=RpwKjlPie68

16
https://www.youtube.com/watch?v=7VS36HTYjEg

17
https://www.youtube.com/watch?v=Hia7XeI1FOw

18
https://www.youtube.com/watch?v=Pcyf1FKLz7A

19
https://www.youtube.com/watch?v=BW8lyRWH7O0

20
https://www.youtube.com/watch?v=kPtxxOHEMWE

21
https://www.youtube.com/watch?v=tT1Kho2ZjoQ

22
https://www.youtube.com/watch?v=GuDC_Zgtfik

23
https://www.youtube.com/watch?v=7jn2dGrku5k

24
https://www.youtube.com/watch?v=VFy7hGS48hE
